# Correction: Engineered human meniscus’ matrix-forming phenotype is unaffected by low strain dynamic compression under hypoxic conditions

**DOI:** 10.1371/journal.pone.0249513

**Published:** 2021-03-29

**Authors:** Alexander R. A. Szojka, Colleen N. Moore, Yan Liang, Stephen H. J. Andrews, Melanie Kunze, Aillette Mulet-Sierra, Nadr M. Jomha, Adetola B. Adesida

The Data Availability statement for this paper is incorrect. The correct statement is: Data are available from http://dx.doi.org/10.17632/4ttbwnsf6z.2.
