# Supplementary figures and images for: Engineered human meniscus’ matrix-forming phenotype is unaffected by low strain dynamic compression under hypoxic conditions
Source: PLoS One. 2021 Mar 10;16(3):e0248292. doi: 10.1371/journal.pone.0248292 (PMC7946300; doi:10.1371/journal.pone.0248292)

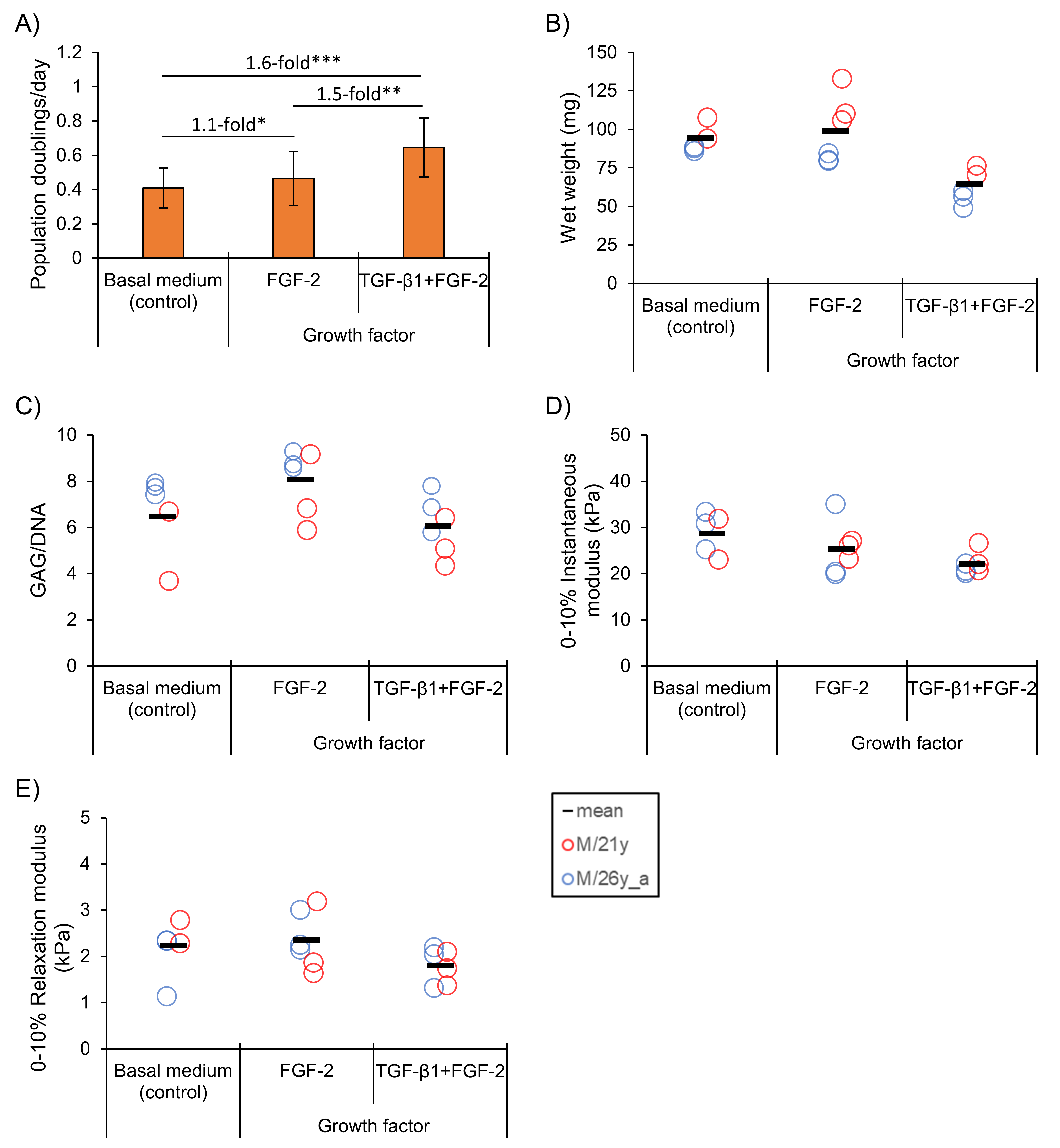

Supplement: S1 Fig — A) All expansion medium contained 10% fetal bovine serum. B-D) Matrix formation was assessed after 3 weeks culture in this preliminary work. [TGF-β1]: 1 ng/mL, [FGF-2]: 5 ng/mL. Differences were assessed by one-way ANOVA. *: p<0.05, **: p<0.01, ***: p<0.001. (TIF) [file pone.0248292.s001.tif]

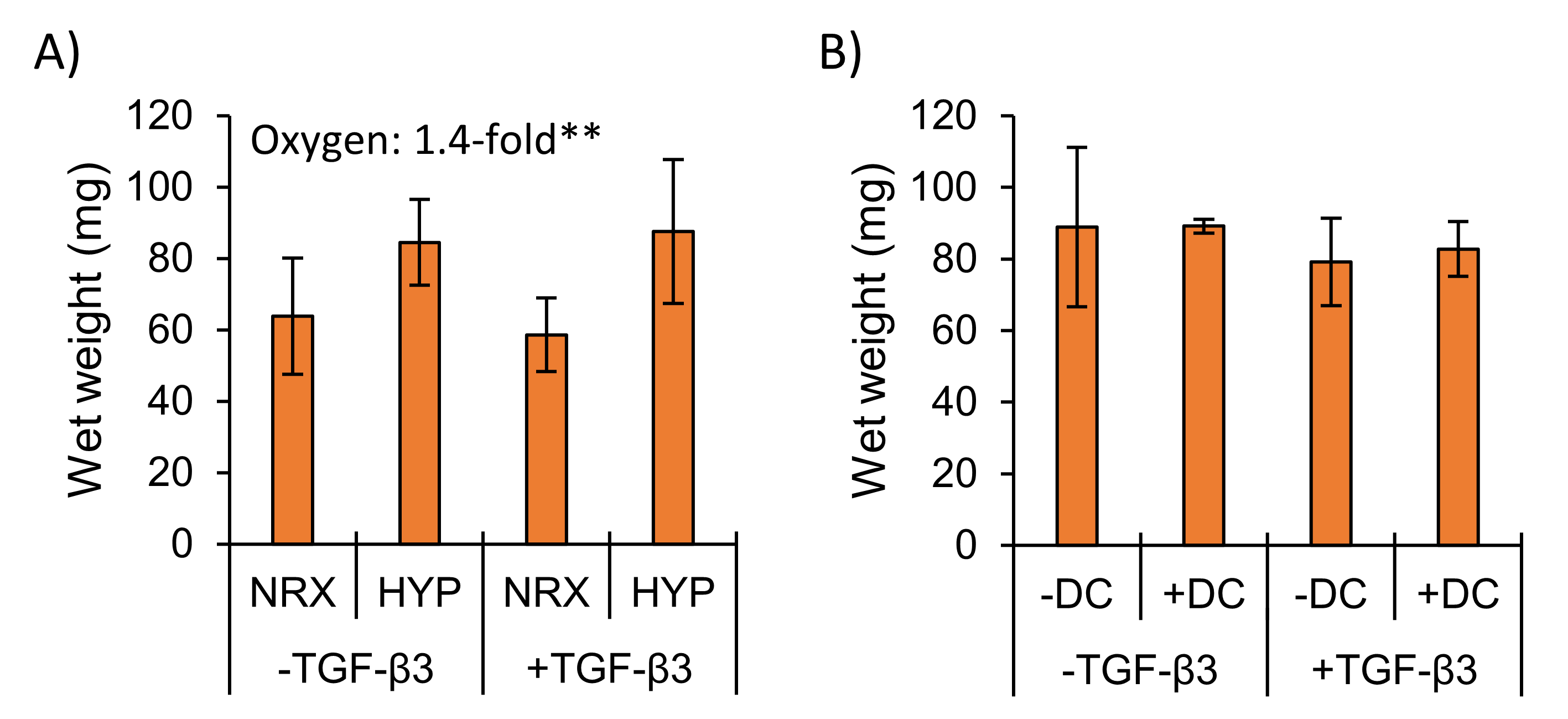

Supplement: S2 Fig — (TIF) [file pone.0248292.s002.tif]

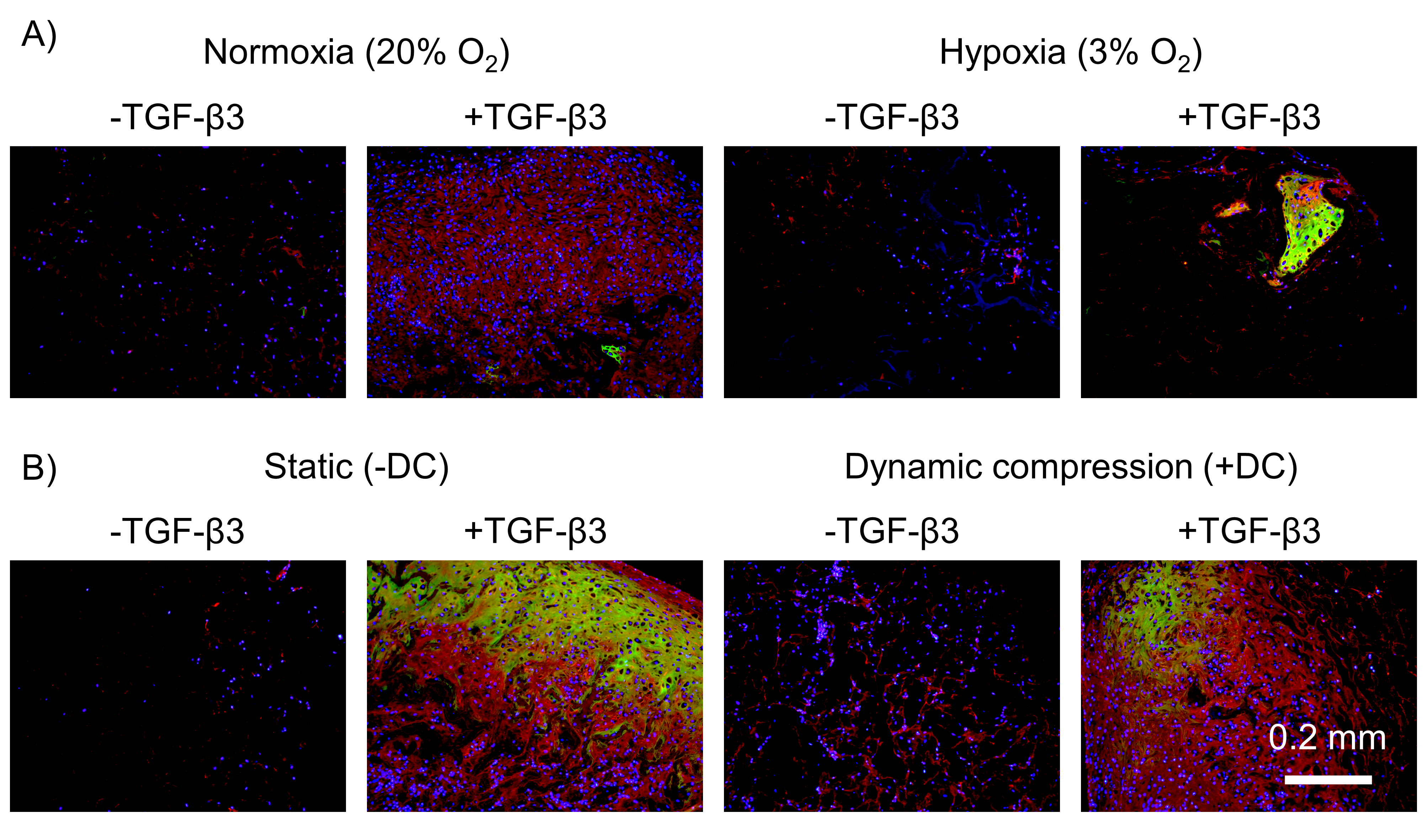

Supplement: S3 Fig — Red: Type I collagen, green: Type II collagen, blue: Cell nuclei by DAPI. All images share a common scale bar. (TIF) [file pone.0248292.s003.tif]

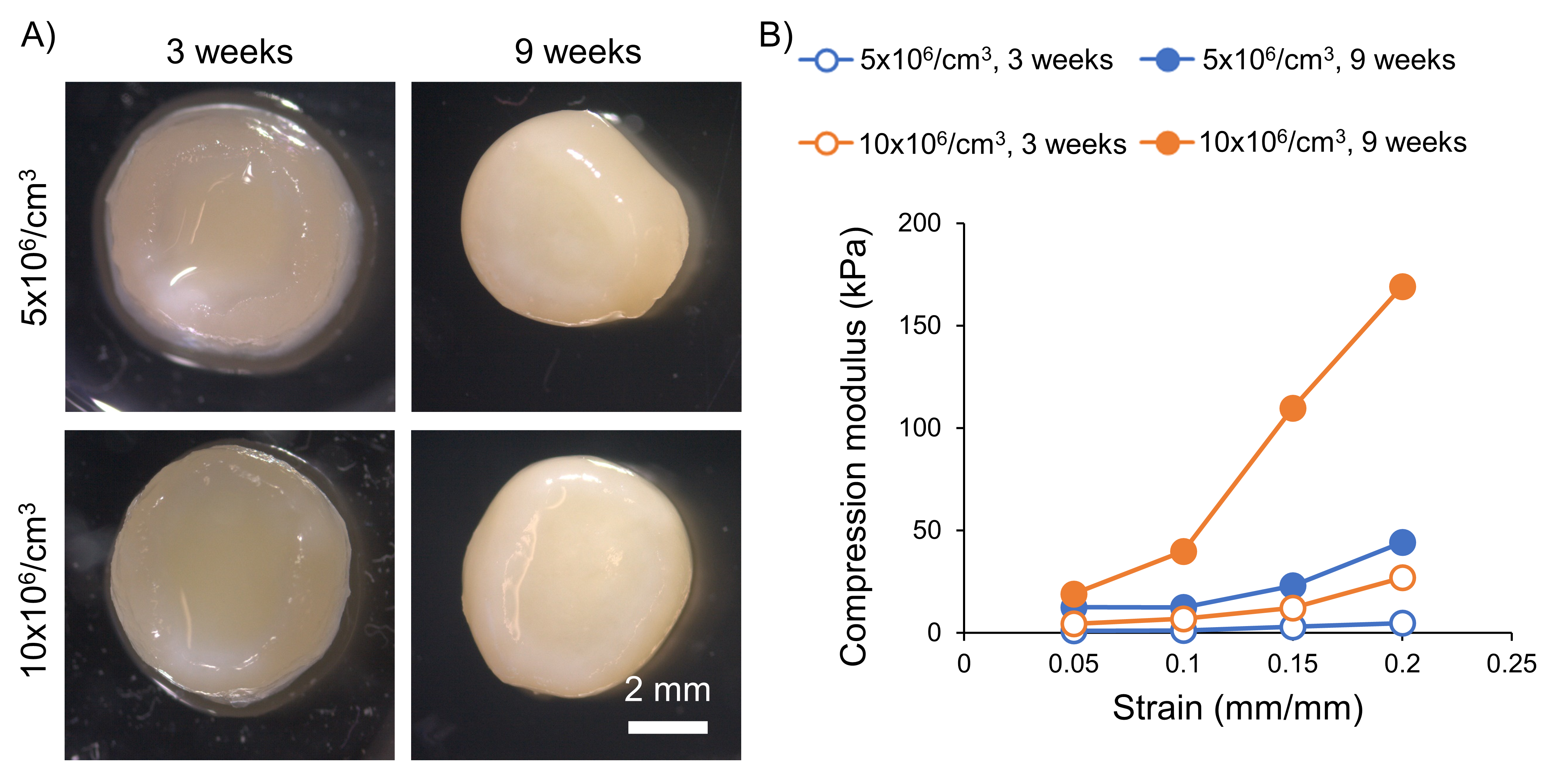

Supplement: S4 Fig — A) Tissue gross morphology at two seeding densities after 3- and 9-weeks’ culture. B) Compression modulus in a ramp test up to 20% strain at 2% strain/minute. This mechanical test was modified from that used in the main study to better show the strain-stiffening behaviour of the tissue constructs. (TIF) [file pone.0248292.s004.tif]
