# Supplementary material for: Engineered human meniscus’ matrix-forming phenotype is unaffected by low strain dynamic compression under hypoxic conditions
Source: PLoS One. 2021 Mar 10;16(3):e0248292. doi: 10.1371/journal.pone.0248292 (PMC7946300; doi:10.1371/journal.pone.0248292)
Supplement: S1 Table — *: The COL1A2 primer described in 10.1089/ten.tea.2019.0306 is preferred for future work. (DOCX) [file pone.0248292.s006.docx]

| **Gene** | **Forward** | **Reverse** | **GenBank Accession** |
| --- | --- | --- | --- |
| *ACAN* | AGGGCGAGTGGAATGATGTT | GGTGGCTGTGCCCTTTTTAC | NM_001135.3 |
| *Β-actin* | AAGCCACCCCACTTCTCTCTAA | AATGCTATCACCTCCCCTGTGT | NM_001101.4 |
| *B2M* | TGCTGTCTCCATGTTTGATGTATCT | TCTCTGCTCCCCACCTCTAAGT | NM_004048.3 |
| *c-FOS* | CGTCTCCAGTGCCAACTTCA | GGTCCGGACTGGTCGAGAT | NM_005252.4 |
| *c-JUN* | CGGAGAGGAAGCGCATGA | TTCCTTTTTCGGCACTTGGA­ | NM_002228.4 |
| *COL1A2** | TTGCCCAAAGTTGTCCTCTTCT | AGCTTCTGTGGAACCATGGAA | AH002625.2 |
| *COL2A1* | CTGCAAAATAAAATCTCGGTGTTCT | GGGCATTTGACTCACACCAGT | NM_001844.5 |
| *COL10A1* | GAAGTTATAATTTACACTGAGGGTTTCAAA | GAGGCACAGCTTAAAAGTTTTAAACA | NM_000493.3 |
| *HIF-1α* | GTAGTTGTGGAAGTTTATGCTAATATTGTGT | TCTTGTTTACAGTCTGCTCAAAATATCTT | NM_001530.4 |
| *HIF-2α* | GGTGGCAGAACTTGAAGGGTTA | GGGCAACACACACAGGAAATC | NM_001430.5 |
| *LOX* | AGGCCACAAAGCAAGTTTCTG | AAATCGCCTGTGGTAGCCATA | NM_002317.7 |
| *SOX9* | CTTTGGTTTGTGTTCGTGTTTTG | AGAGAAAGAAAAAGGGAAAGGTAAGTTT | NM_000346.3 |
| *TGF-β1* | GGGAAATTGAGGGCTTTCG | AGTGTGTTATCCCTGCTGTCACA | NM_003239.4 |
| *TGF-β3* | CTGGCCCTGCTGAACTTTG | AAGGTGGTGCAAGTGGACAGA | NM_003239.4 |
| *VEGF* | GCACGGTCCCTCTTGGAA | CGGTGATTTAGCAGCAAGAAAA | NM_001025366.3 |
| *YWHAZ* | TCTGTCTTGTCACCAACCATTCTT | TCATGCGGCCTTTTTCCA | NM_003406.3 |
