# Supplementary material for: Engineered human meniscus’ matrix-forming phenotype is unaffected by low strain dynamic compression under hypoxic conditions
Source: PLoS One. 2021 Mar 10;16(3):e0248292. doi: 10.1371/journal.pone.0248292 (PMC7946300; doi:10.1371/journal.pone.0248292)
Supplement: Attachment — Submitted filename: Szojka et.al. PlosOne 2020.docx [file pone.0248292.s007.docx]

Alexander R.A. Szojka et. al. PLOS One:

Engineered human meniscus' matrix-forming phenotype is unaffected by low strain dynamic compression under hypoxic condition.

Comments:

The authors seem to have extended their previously published work in the Tissue Engg. Part A. 2019- Hypoxia and TGF-b3 Synergistically Mediate Inner Meniscus-Like Matrix Formation by Fibrochondrocytes

It is good study and fills the gap in our understanding on meniscus matrix-forming ability under dynamic compression. However, since majority of the findings were already established in their previous work, specifically the role of TGF-beta3 in hypoxic conditions backed by good experimental evidence, I feel the current work doesn’t add significant information. It can be a part of the paper but cannot be considered a full paper. I advise authors can consider expanding their study further and resubmit it. Also, they will need to provide a novelty statement in context of their previously published work.
