# Supplementary material for: Engineered human meniscus’ matrix-forming phenotype is unaffected by low strain dynamic compression under hypoxic conditions
Source: PLoS One. 2021 Mar 10;16(3):e0248292. doi: 10.1371/journal.pone.0248292 (PMC7946300; doi:10.1371/journal.pone.0248292)
Supplement: Attachment — Submitted filename: Response to Reviewers.docx [file pone.0248292.s009.docx]

**Response to Reviewers**

Alex Szojka, February 17, 2021

We thank the reviewers and editor for their time in reviewing our work and address the outstanding comment below.

Reviewer #2: The authors claim in the conclusion section seems incorrect. Line 438-442: The present study demonstrates that, in a type I collagen scaffold seeded with MFCs from partial meniscectomy, neither continuous hypoxia under static conditions nor hypoxia combined with dynamic compression to 10% strain induced a fibrocartilaginous inner meniscus-like matrix-forming phenotype, regardless of TGF-beta supplementation. From their data, TGF beta is indeed an important factor to induce fibrocartilaginous matrix formation! They need to clarify.

Thank you for identifying this problem with our original text. We realize now that the original writing (“regardless of TGF-β3 supplementation”) failed to make the distinction below:

- Hypoxia and hypoxia with dynamic compression did not ***induce*** a fibrocartilaginous phenotype.
- Hypoxia and hypoxia with dynamic compression did not ***promote*** the fibrocartilaginous phenotype when it was induced by TGF-β3 supplementation

Thus, we have now clarified the concluding text:

The present study demonstrates that, in a type I collagen scaffold seeded with MFCs from partial meniscectomy, neither continuous hypoxia under static conditions nor hypoxia combined with dynamic compression to 10% strain induced a fibrocartilaginous inner meniscus-like matrix-forming phenotype in the absence of TGF-β3 supplementation. They also did not promote the inner meniscus-like matrix-forming phenotype when it was induced by TGF-β3 supplementation.
